# Loss of endothelial TRPC1 aggravates metabolic dysfunction in obesity via disrupting adipose tissue homeostasis

**DOI:** 10.3389/fmolb.2025.1619559

**Published:** 2025-06-11

**Authors:** Yihui Lan, Shiquan Wang, Yuan Chu, Yizhi Zhang, Yuan Liu, Fan Yu, Lei Feng, Yifei Zhu

**Affiliations:** Wuxi School of Medicine, Jiangnan University, Wuxi, China

**Keywords:** TRPC1, endothelial cells, obesity, metabolic dysfunction, inflammation, thermogenesis, metabolomics

## Abstract

**Introduction:**

While obesity exacerbates metabolic disorders through vascular endothelial dysfunction, the specific regulatory mechanisms of endothelial cells underlying this process remain poorly defined. Although the transient receptor potential canonical 1 (TRPC1) channel demonstrates tissue-specific heterogeneity in metabolic regulation, its functional role within endothelial cells and its contribution to metabolic disturbances associated with obesity remain unresolved.

**Methods:**

We established endothelial-specific TRPC1 knockout (TRPC1_EC_^−/−^) and overexpression (TRPC1_EC_^KI/KI^) mouse models, which were integrated with a high-fat diet (HFD)-induced obesity paradigm. Through comprehensive metabolic phenotyping, adipose tissue molecular profiling, and serum metabolomics analysis, we systematically dissected the regulatory mechanisms of endothelial TRPC1 in glucose and lipid metabolism.

**Results:**

Endothelial TRPC1 deficiency, while not altering the severity of HFD-induced obesity, significantly exacerbates impaired glucose tolerance, insulin resistance, and dyslipidemia. Mechanistically, the deficiency of endothelial TRPC1 enhances the expression of chemokines (CCL3/CXCL5) and pro-inflammatory cytokines (IL-1β/TIMP1), thereby creating an inflammatory microenvironment in epididymal white adipose tissue (eWAT) and suppressing PGC1α/UCP1-mediated thermogenic function. Metabolomic profiling further reveals that TRPC1 deficiency drives systemic metabolic perturbations, including the depletion of serum 1-methylhistidine and N-acetylvaline, alongside the aberrant accumulation of gibberellin A12, which suggests disrupted amino acid metabolism and the activation of non-canonical inflammatory pathways. Conversely, endothelial TRPC1 overexpression significantly ameliorates obesity-associated metabolic dysfunction, as evidenced by reduced visceral fat deposition, enhanced insulin sensitivity, and restored thermogenic capacity in adipose tissue.

**Conclusion:**

This study, for the first time, elucidates the pivotal role of endothelial TRPC1 in maintaining metabolic homeostasis by orchestrating an “inflammation-thermogenesis-metabolite” regulatory network. Specifically, the deficiency of endothelial TRPC1 exacerbates metabolic dysfunction associated with obesity, whereas its overexpression exerts significant protective effects. These findings highlight the centrality of endothelial ion channels in vascular-metabolic coupling, thereby establishing a theoretical rationale for targeting TRPC1 as a therapeutic strategy against metabolic syndrome.

## 1 Introduction

Obesity, recognized as a global public health crisis, is pathologically characterized by systemic metabolic disturbances resulting from excessive fat accumulation (Hruby and Hu, 2014). Notably, vascular endothelial dysfunction significantly increases the morbidity and mortality risks associated with cardiovascular diseases and type 2 diabetes through mechanisms involving insulin resistance, chronic inflammation, and dysregulated lipid metabolism (Leguina-Ruzzi et al., 2015; Huang et al., 2016; Al-Salameh et al., 2019; Cabral et al., 2023). Therefore, elucidating the regulatory mechanisms of the vascular system in the pathogenesis of obesity is a critical avenue for developing innovative therapeutic strategies to combat metabolic disorders.

Within the systemic metabolic disturbances induced by obesity, endothelial cells, due to their strategic positioning at the vascular interface, exert critical regulatory functions (Sturtzel, 2017). These cells dynamically regulate hemodynamic equilibrium through calcium-dependent vasomotor responses while precisely coordinating peripheral glucose uptake and glycogen metabolism via the GLUT1-mediated PI3K-Akt signaling axis, thus maintaining systemic glucose-lipid homeostasis (De Nigris et al., 2015; Sáez et al., 2018). During the pathogenesis of obesity, adipose tissue-derived TNF-α and IL-6 activate the NF-κB signaling cascade, inducing aberrant expression of endothelial adhesion molecules and driving monocyte infiltration, which establishes a pro-inflammatory perivascular microenvironment (Asokan et al., 2018; Aladhami et al., 2021). This pathological sequence culminates in endothelial dysfunction characterized by eNOS inactivation-mediated vasodilatory impairment, increased vascular permeability through tight junction protein degradation, and RhoA-ROCK pathway-dependent vasoconstriction—all converging to suppress insulin receptor tyrosine phosphorylation and precipitate systemic insulin resistance (Boido et al., 2015; Cat et al., 2018; Kiyooka et al., 2022). Such endothelial dysfunction establishes a mechanistic nexus between biomechanical abnormalities and metabolic derangements, solidifying its central role in obesity-related comorbidities.

As a member of the transient receptor potential canonical (TRPC) subfamily of non-selective cation channels, transient receptor potential canonical 1 (TRPC1) exhibits tissue-specific functions in metabolic regulation (Wang H. et al., 2020). TRPC1 is highly expressed in brown adipose tissue (BAT), where it modulates metabolic gene expression, respiratory function, and thermoregulation. A deficiency in TRPC1 results in increased body weight and impaired metabolic control in mice (Wolfrum et al., 2018). In the regulation of insulin secretion, TRPC1 acts as a component of store-operated Ca^2+^ channels, forming a functional complex with Orai1 that is regulated by Stromal Interaction Molecule 1 (STIM1). This complex plays a well-defined role in glucose-stimulated insulin secretion (Sabourin and Allagnat, 2016). Furthermore, in adipose tissue, TRPC1 deficiency inhibits adipocyte differentiation, reduces the secretion of adiponectin and leptin, and disrupts metabolic homeostasis (Schaar et al., 2019). However, the metabolic regulatory role of endothelial TRPC1—particularly its systemic impact on energy homeostasis under obese conditions—remains largely undefined.

This study constructed mouse models with endothelial cell-specific TRPC1 deficiency and overexpression to investigate their roles in obesity-related metabolic disorders. Our findings demonstrate that endothelial TRPC1 deficiency exacerbates insulin resistance induced by a high-fat diet and disrupts glucose and lipid metabolism. Importantly, endothelial-specific TRPC1 overexpression reverses metabolic dysfunction associated with obesity. These results reveal, for the first time, the critical role of endothelial TRPC1 in energy homeostasis through its regulation of vascular-metabolic coupling mechanisms, thereby providing experimental evidence to support the therapeutic targeting of endothelial ion channels in metabolic syndrome.

## 2 Materials and methods

### 2.1 Animals

All animal experiments adhered to the NIH Guidelines for the Care and Use of Laboratory Animals and received ethical approval from the Jiangnan University Institutional Animal Care and Use Committee (IACUC; Approval No.: JN.NO20220415C0040930[116]). Mice were housed in the Experimental Animal Center of Wuxi School of Medicine under standardized conditions (26°C, 12-h light/dark cycle, *ad libitum* access to food and water). Only male mice were used in this study. TRPC1-flox/flox, TRPC1-KI/flox, and EC-Cre mice (C57BL/6J background) were acquired from the Jiangsu Institute of Medical Innovation. Endothelial-specific TRPC1 knockout (TRPC1_EC_^−/−^) and knockin (TRPC1_EC_^KI/KI^) models were generated by crossing TRPC1-flox/flox or TRPC1-KI/flox mice with EC-Cre mice, using littermate TRPC1^fl/fl^ mice as controls. At 6°weeks of age, all mice received intraperitoneal tamoxifen injections (20 mg/kg; Macklin Biochemical, China) daily for 7 consecutive days to induce tissue-specific gene deletion/overexpression, followed by a 1-week recovery period. To ensure experimental consistency, male mice were used in all experiments, and all groups received Tamoxifen administration (Krüger et al., 2020). Obesity was induced in all mice via 12-week high-fat diet (HFD) feeding (45% kcal from fat; Nantong Trophic, China). At study termination, euthanasia was performed by carbon dioxide asphyxiation followed by cervical dislocation.

### 2.2 Genotyping of mice

Genomic DNA was extracted from mouse tail biopsies using a commercial DNA isolation kit (Yeasen Biotechnology, China). The primer sequences, as listed in Supplementary Table S1, were utilized for the PCR amplification of genomic DNA. The PCR amplification protocol is detailed in Supplementary Tables S2–S4. The PCR products were resolved through agarose gel electrophoresis and visualized using a Tanon-5200 Multi Gel Imaging System (China).

### 2.3 Immunofluorescence staining

Thoracic aortic frozen sections were fixed in 4% paraformaldehyde (Beyotime, China), rinsed with phosphate-buffered saline (PBS), permeabilized with 0.1% Triton X-100, and subsequently blocked with 5% bovine serum albumin (BSA). The sections were incubated overnight at 4°C with primary antibodies: anti-TRPC1 (1:200, Santa Cruz Biotechnology) and anti-CD31 (1:200, Santa Cruz Biotechnology). Secondary labeling was conducted using Alexa Fluor 568-conjugated goat anti-rabbit (1:200, Invitrogen) and Alexa Fluor 647-conjugated goat anti-mouse (1:200, Invitrogen) antibodies. For nuclear counterstaining, DAPI (1:1,000, Beyotime) was employed. Immunofluorescence imaging was performed using a Zeiss LSM 880 confocal microscope (Germany).

### 2.4 Micro-CT imaging of mice

Adipose tissue distribution was analyzed using the IVIS SPECTRUM *in vivo* micro-CT system (PerkinElmer, United States). Image analysis effectively differentiated between adipose tissue depots, with visceral adipose tissue represented in yellow and subcutaneous adipose tissue depicted in green. Volumetric quantification of the designated regions was subsequently performed using proprietary analysis software.

### 2.5 Comprehensive respiratory and metabolic monitoring

Respiratory and metabolic parameters were continuously monitored using the Comprehensive Laboratory Animal Monitoring System (CLAMS; Columbus Instruments, United States) at the Jiangnan University Experimental Animal Center. Following a 2-day acclimation period, metabolic phenotyping was conducted over three consecutive days. The recorded parameters included oxygen consumption (VO_2_), carbon dioxide production (VCO_2_), energy expenditure, respiratory exchange ratio (RER), water and food intake, and locomotor activity.

### 2.6 Glucose and insulin tolerance tests

Glucose tolerance tests (GTT) were conducted after a 12-h fasting period, during which intraperitoneal glucose was administered at a dosage of 1.5 g/kg (Solarbio, China). For insulin tolerance tests (ITT), mice were subjected to a 4-h fasting period prior to receiving an intraperitoneal insulin injection at a dosage of 0.75 U/kg (Beyotime, China). Blood glucose levels were monitored at specified time points using a glucometer (Yuwell Medical, China), with blood samples collected from the tail vein.

### 2.7 Lipid profile analysis and serum metabolomics

Serum levels of total cholesterol (TC), triglycerides (TG), low-density lipoprotein cholesterol (LDL-C), and high-density lipoprotein cholesterol (HDL-C) were measured using commercial kits from Nanjing Jiancheng Bioengineering Institute (China), strictly adhering to the manufacturer’s protocols. Additionally, serum metabolomic profiling was conducted by BGI Genomics (China).

### 2.8 Hematoxylin and eosin (H&E) staining

Fresh tissue specimens were fixed in 4% paraformaldehyde for 24 h, rinsed with running water for 1 h, and subsequently immersed in PBS for 30 min. The tissues underwent gradient ethanol dehydration (75% ethanol for 4 h, 85% ethanol for 2 h, 90% ethanol for 2 h, and 95% ethanol for 1 h, followed by two changes of absolute ethanol for 30 min each), xylene clearing (three cycles of 20 min each), and paraffin infiltration (three cycles of 1 h each). Paraffin-embedded tissues were sectioned to a thickness of 4 μm and stained using H&E kits (Solarbio, China). Histological images were acquired using the Pannoramic MiDi slide scanner system (3DHISTECH, Hungary). Morphometric analysis of adipocyte size distribution in adipose tissue was conducted using ImageJ software. Adipocytes were stratified into three categories based on equivalent diameter (<50 μm, 50–100 μm, and >100 μm) through automated segmentation of histological sections. Size distribution profiles were generated by quantifying the proportional representation of each cellular cohort (Stenkula and Erlanson-Albertsson, 2018; Wang S. et al., 2020).

### 2.9 RNA extraction and quantitative PCR (qPCR)

Total RNA was isolated from tissues using an RNA extraction kit (Yeasen, China). Complementary DNA (cDNA) synthesis was performed with the QuantiTect Reverse Transcription Kit (Yeasen, China). Quantitative PCR (qPCR) assays were conducted on a LightCycler 480 system (Roche, Switzerland) utilizing Hieff UNICON qPCR SYBR Green Master Mix (Yeasen, China). The primer sequences are detailed in Supplementary Table S5. All reactions were conducted in technical triplicate, with TATA-binding protein (TBP) serving as the reference gene (Ye et al., 2012). Relative gene expression was quantified using the 2^−ΔΔCt^ method.

### 2.10 Western blot analysis

Total protein was extracted using RIPA Lysis Buffer (Beyotime Biotechnology, China) supplemented with phenylmethylsulfonyl fluoride (PMSF). Protein samples were resolved via 10% sodium dodecyl sulfate-polyacrylamide gel electrophoresis (SDS-PAGE) and subsequently transferred onto polyvinylidene fluoride (PVDF) membranes (Beyotime Biotechnology, China). Membranes were blocked with 5% bovine serum albumin (BSA) for 2 h at room temperature, followed by overnight incubation at 4°C with primary antibodies: anti-UCP1 (1:1,000, Cell Signaling Technology, CST), anti-PGC1α (1:200, Santa Cruz Biotechnology), and anti-β-tubulin (1:5000, CST). After washing to remove unbound primary antibodies, membranes were incubated with horseradish peroxidase (HRP)-conjugated secondary antibodies (Beyotime Biotechnology, China) for 1 h at room temperature. Protein bands were visualized and quantified using ImageJ software (National Institutes of Health, NIH, USA).

### 2.11 Quantification and statistical analysis

Experimental data are presented as mean ± standard error of the mean (SEM), with n ≥ 3 biological replicates. Statistical analyses were performed using GraphPad Prism 8.0 software (GraphPad Software, United States). Time-course response curves were analyzed using repeated measures two-way analysis of variance (ANOVA), followed by Bonferroni *post hoc* tests. Comparisons between two groups were conducted using unpaired two-tailed Student’s t-tests. For comparisons involving three or more groups, one-way ANOVA with Tukey’s multiple comparisons test was applied. Statistical significance was defined as *p* < 0.05.

## 3 Results

### 3.1 Endothelial TRPC1 deficiency does not affect the development of obesity

**FIGURE 1:**
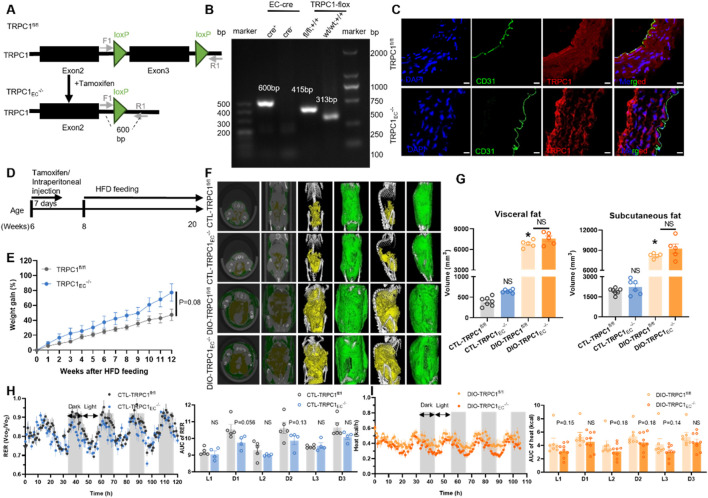
Endothelial TRPC1 deficiency does not affect the development of obesity. **(A)** Schematic of TRPC1_EC_^−/−^ mouse construction via endothelial cell (EC)-specific Cre-LoxP recombination. **(B)** Genotyping PCR of TRPC1_EC_^−/−^ and wild-type mice. **(C)** Immunofluorescence staining of thoracic aorta in TRPC1^fl/fl^ and TRPC1_EC_^−/−^ mice: nuclei (DAPI, blue), endothelial marker (CD31, green), and TRPC1 (red). Scale bar: 10 μm. **(D)** Experimental design for high-fat diet (HFD)-induced obesity model. **(E)** Body weight trajectories of HFD-fed mice (n = 4–5/group). **(F)** Representative micro-CT images of adipose tissue distribution in CTL-TRPC1^fl/fl^, CTL-TRPC1_EC_^−/−^, DIO-TRPC1^fl/fl^, and DIO-TRPC1_EC_^−/−^ mice (subcutaneous fat: green; visceral fat: yellow). **(G)** Quantitative micro-CT analysis of subcutaneous and visceral fat volumes across groups (n = 5–7/group). **(H)** Respiratory exchange ratio (RER) dynamics in CTL mice, quantified by area under the curve (AUC; n = 4–5/group). **(I)** Heat production of DIO mice via AUC analysis of energy expenditure phases (n = 7–9/group). NS: Not significant (*p* > 0.05).

Building on the critical role of TRPC1 in cardiovascular homeostasis and the pathogenesis of obesity, this study investigates its functional mechanisms in endothelial metabolic regulation. Our previous research has demonstrated that TRPC1 protein expression in endothelial cells is significantly downregulated under obese conditions, and that endothelial-specific TRPC1 deficiency results in impaired vascular contractility and dysregulated blood pressure regulation (Zhu et al., 2025). To elucidate the metabolic regulatory functions of endothelial TRPC1, we generated a tamoxifen-inducible, endothelial cell-specific TRPC1 knockout mouse model (TRPC1_EC_^−/−^, Figure 1A). Genomic PCR analysis confirmed the successful Cre-loxP-mediated recombination specifically in the endothelial cells of TRPC1_EC_^−/−^ mice, as evidenced by the characteristic 600 bp amplification product (Figure 1B). Immunofluorescence staining analysis of thoracic aorta sections further demonstrated the complete ablation of TRPC1 signaling in the endothelial layer of TRPC1_EC_^−/−^ mice (Figure 1C), thereby validating the efficiency of the endothelial-specific knockout.

To investigate the metabolic regulatory functions of endothelial TRPC1 under both physiological and obese conditions, we employed a high-fat diet (HFD)-induced obesity model. Eight-week-old TRPC1^fl/fl^ and TRPC1_EC_^−/−^ mice were subjected to a 12-week isocaloric HFD intervention (Figure 1D). Longitudinal monitoring revealed no significant differences in weight gain trajectory or final body weight at 20 weeks of age between the two groups (Figure 1E; Supplementary Figure S1A). A detailed assessment of adipose tissue distribution through micro-computed tomography analysis demonstrated comparable pathological expansion of both visceral and subcutaneous adipose tissue volumes following HFD feeding, with no significant differences observed between genotypes (Figures 1F,G).

To evaluate the impact of endothelial TRPC1 deficiency on basal metabolic activity, we conducted comprehensive respiratory metabolic monitoring of experimental animals. Under basal metabolic conditions, CTL-TRPC1_EC_^−/−^ mice exhibited a decreasing trend in the RER compared to CTL-TRPC1^fl/fl^ controls; however, this difference did not reach statistical significance (Figure 1H). No intergroup differences were observed in parameters such as food intake, locomotor activity, or heat production (Supplementary Figures S1B–I). Under obese conditions, DIO-TRPC1_EC_^−/−^ mice showed reduced heat production compared to their DIO-TRPC1^fl/fl^ counterparts, but this effect also failed to achieve statistical significance (Figure 1I). Other metabolic parameters, including RER, food intake, and physical activity, remained stable between the obese genotypes (Supplementary Figures S1J–Q). Collectively, these findings indicate that endothelial TRPC1 deficiency does not alter the development of HFD-induced obesity; however, it may contribute to metabolic regulation through non-significantly reduced energy expenditure.

### 3.2 Endothelial TRPC1 deficiency exacerbates obesity-induced dysregulation of glucose and lipid metabolism

**FIGURE 2:**
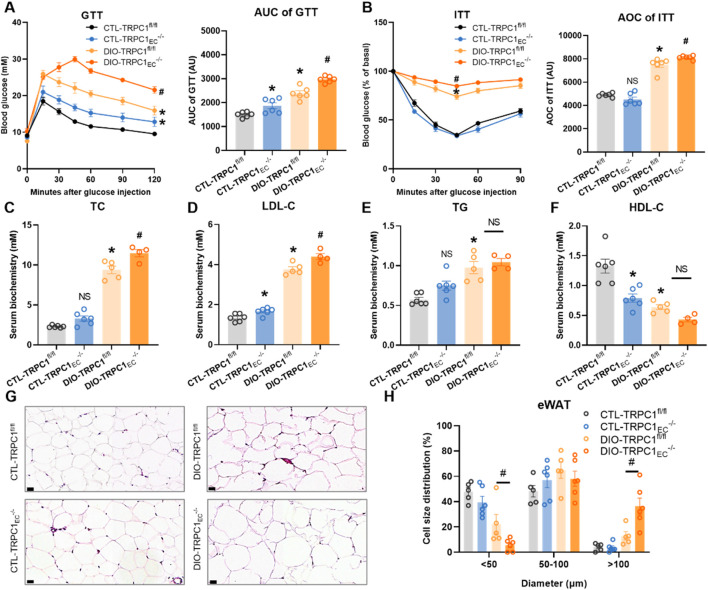
Endothelial TRPC1 deficiency exacerbates obesity-induced dysregulation of glucose and lipid metabolism. **(A)** Intraperitoneal glucose tolerance test (GTT, 1.5 g/kg) with area under the curve (AUC) analysis for all groups (n = 6/group). **(B)** Intraperitoneal insulin tolerance test (ITT, 0.75 U/kg) with AUC quantification (n = 6/group). **(C–F)** Serum levels of total cholesterol (TC), low-density lipoprotein cholesterol (LDL-C), triglycerides (TG), and high-density lipoprotein cholesterol (HDL-C) in four groups (n = 4–6/group). **(G)** Representative hematoxylin and eosin, (H&E) staining of epididymal white adipose tissue (eWAT) in four groups. Scale bar: 20 μm. **(H)** Size distribution pattern of adipocytes in eWAT in four groups (n = 5–6/group). *p < 0.05 vs. CTL-TRPC1^fl/fl^; ^*#*^ *p* < 0.05 vs. DIO-TRPC1^fl/fl^; NS: Not significant (*p* > 0.05).

To further elucidate the role of endothelial TRPC1 deficiency in the pathogenesis of obesity, we systematically evaluated the glucose and lipid metabolic profiles in TRPC1_EC_^−/−^ and TRPC1^fl/fl^ mice under both basal and obese conditions. Intraperitoneal glucose tolerance tests revealed that CTL-TRPC1_EC_^−/−^ mice exhibited significantly impaired glucose clearance compared to CTL-TRPC1^fl/fl^ controls under basal conditions. However, insulin tolerance tests showed no genotype-specific differences (Figures 2A,B). In contrast, obese DIO-TRPC1_EC_^−/−^ mice demonstrated more severe glucose intolerance and insulin resistance compared to their DIO-TRPC1^fl/fl^ counterparts (Figures 2A,B). Lipid metabolic analysis indicated that HFD-induced dyslipidemia was present in TRPC1^fl/fl^ mice, characterized by elevated serum total TC, LDL-C, and TG, alongside a concurrent reduction in HDL-C (Figures 2C–F). Notably, endothelial TRPC1 deficiency exacerbated these lipid abnormalities under obese conditions: DIO-TRPC1_EC_^−/−^ mice displayed significantly increased levels of TC, LDL-C, and TG, along with further reductions in HDL-C compared to DIO-TRPC1^fl/fl^ mice (Figures 2C–F), indicating exacerbated dyslipidemia. In the context of obesity, insulin resistance and dyslipidemia frequently arise from impaired adipose tissue function (Choi et al., 2021). Our histopathological analysis of adipose tissues demonstrated that endothelial TRPC1 deficiency exacerbates high-fat diet-induced epididymal white adipose tissue (eWAT) hypertrophy, resulting in an increased proportion of large adipocytes and a concomitant reduction in small adipocytes within adipose depots, while no significant differences were observed in inguinal white adipose tissue (iWAT) or brown adipose tissue (BAT) (Figures 2G,H; Supplementary Figures S2A,B). These findings suggest that endothelial TRPC1 deficiency drives adipocyte hypertrophy in eWAT, leads to aberrant lipid deposition in serum, disrupts glucose and lipid metabolic homeostasis, and ultimately contributes to systemic insulin resistance.

### 3.3 Endothelial TRPC1 deficiency exacerbates adipose tissue inflammation and impairs thermogenic gene expression

**FIGURE 3:**
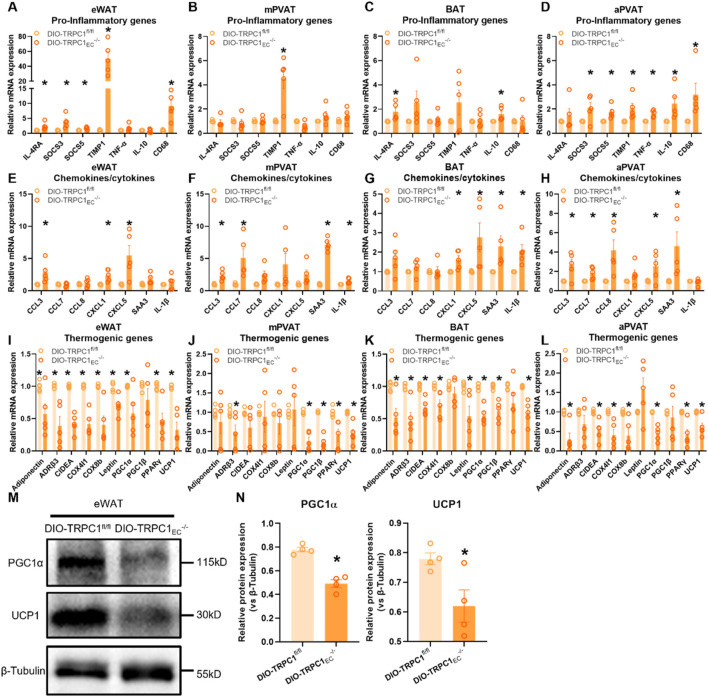
Endothelial TRPC1 deficiency exacerbates adipose tissue inflammation and impairs thermogenic gene expression. **(A–D)** Relative mRNA expression of pro-inflammatory genes in eWAT, mesenteric perivascular adipose tissue (mPVAT), brown adipose tissue (BAT), and aortic perivascular adipose tissue (aPVAT) from DIO-TRPC1^fl/fl^ and DIO-TRPC1_EC_^−/−^ mice (n = 4–5/group). **(E–H)** Relative mRNA levels of chemokines/cytokines in the same tissues and mouse groups (n = 4–5/group). **(I–L)** Relative mRNA expression of thermogenic genes in indicated tissues (n = 4–5/group). **(M,N)** Representative western blots and quantitative analysis of PGC1α and UCP1 protein levels in eWAT lysates from DIO-TRPC1^fl/fl^ and DIO-TRPC1_EC_^−/−^ mice (n = 4–5/group). *p < 0.05 vs. DIO-TRPC1^fl/fl^ group.

Adipose tissue plays a pivotal role in regulating energy homeostasis and metabolic balance. However, an imbalance in its inflammatory microenvironment contributes to metabolic disorders, including insulin resistance (Kunz et al., 2021). Through a systematic molecular characterization of distinct adipose depots in endothelial-specific TRPC1 knockout mice, this study elucidates the critical role of endothelial TRPC1 in modulating adipose tissue inflammation and metabolic function. Notably, we observed a significant upregulation of tissue inhibitor of metalloproteinases 1 (TIMP1) in eWAT and mesenteric perivascular adipose tissue (mPVAT), while brown adipose tissue (BAT) and aortic perivascular adipose tissue (aPVAT) exhibited aberrant elevations of interleukin-10 (IL-10) (Figures 3A–D). Chemokine profiling revealed marked increases in serum amyloid A3 (SAA3) levels in mPVAT, BAT, and aPVAT, accompanied by significantly enhanced expression of CC-chemokine ligand 3 (CCL3) and CXC-chemokine ligand 5 (CXCL5) in eWAT and aPVAT. Of particular interest, the concurrent upregulation of IL-1β in BAT and mPVAT suggests differential inflammatory regulatory mechanisms across adipose depots (Figures 3E–H).

At the metabolic functional level, DIO-TRPC1_EC_^−/−^ mice exhibit systemic thermogenic impairment. The transcriptional and protein expression levels of peroxisome proliferator-activated receptor gamma coactivator 1-alpha (PGC1α) and uncoupling protein 1 (UCP1)—key regulators of thermogenesis—are significantly reduced across all examined adipose depots (Figures 3I–N). PGC1α, a master regulator of mitochondrial biogenesis and oxidative metabolism, directly influences the thermogenic capacity of brown adipose tissue, while UCP1 dysfunction, as the mitochondrial inner membrane thermogenic effector, exacerbates energy metabolic defects (Shen et al., 2021; Rahbani et al., 2024). Notably, endothelial TRPC1 deficiency recapitulates these thermogenic defects and pro-inflammatory gene expression patterns under basal conditions (Supplementary Figure S3), although with greater severity in obese states. Mechanistically, TRPC1-deficient endothelial cells promote chemokine-mediated immune cell infiltration and local inflammatory responses, which in turn suppress adipocyte thermogenic function through paracrine inflammatory signaling. This pathological cascade ultimately contributes to metabolic phenotypes characterized by glucose intolerance and systemic insulin resistance.

### 3.4 Serum metabolomic alterations induced by endothelial TRPC1 deficiency

**FIGURE 4:**
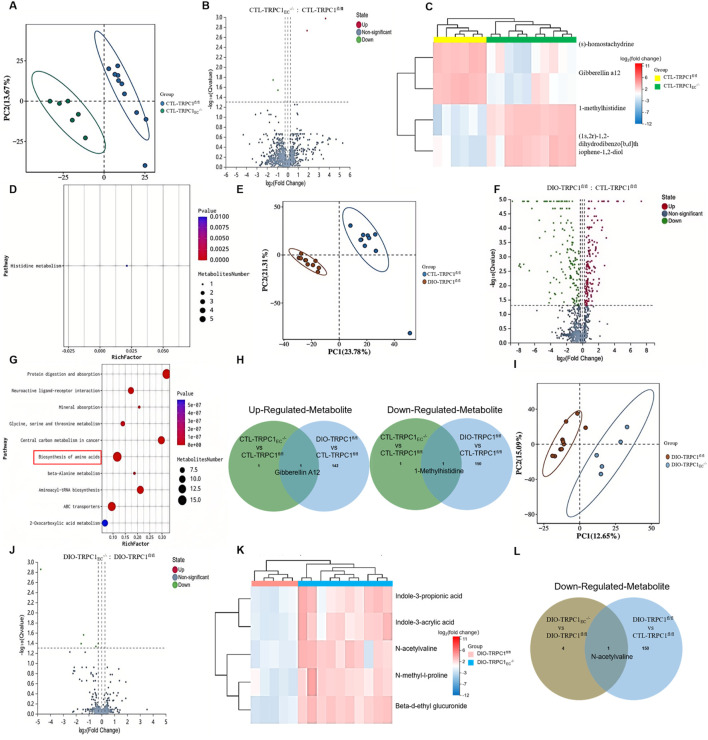
Serum metabolomic alterations induced by endothelial TRPC1 deficiency. **(A)** Principal component analysis (PCA) separating serum samples from CTL-TRPC1_EC_^−/−^(green) and CTL-TRPC1^fl/fl^ (blue) mice (n = 6–10/group). **(B)** Volcano plot highlighting significantly altered metabolites between CTL-TRPC1_EC_^−/−^ and CTL-TRPC1^fl/fl^ groups. **(C)** Correlation heatmap of serum metabolites in CTL-TRPC1_EC_^−/−^ versus CTL-TRPC1^fl/fl^ mice. **(D)** Metabolite set enrichment analysis identifying disrupted histidine metabolism in CTL-TRPC1_EC_^−/−^ mice. **(E)** PCA distinguishing DIO-TRPC1^fl/fl^ (brown) and CTL-TRPC1^fl/fl^ (blue) serum profiles (n = 9–10/group). **(F)** Volcano plot of differentially abundant metabolites between DIO-TRPC1^fl/fl^ and CTL-TRPC1^fl/fl^ groups. **(G)** Pathway enrichment analysis of all significantly altered metabolites in DIO vs. CTL comparison. **(H)** Venn diagram illustrating overlapping and unique upregulated/downregulated metabolites in CTL-TRPC1_EC_^−/−^ vs. CTL-TRPC1^fl/fl^ and DIO-TRPC1^fl/fl^ vs. CTL-TRPC1^fl/fl^ comparisons. **(I)** PCA separating serum samples from DIO-TRPC1_EC_^−/−^ (blue) and DIO-TRPC1^fl/fl^ (brown) mice (n = 5–10/group). **(J)** Volcano plot emphasizing significant metabolite alterations in DIO-TRPC1_EC_^−/−^ vs. DIO-TRPC1^fl/fl^ comparison. **(K)** Correlation heatmap of serum metabolites in DIO-TRPC1_EC_^−/−^ versus DIO-TRPC1^fl/fl^ mice. **(L)** Venn diagram showing commonly downregulated metabolites in DIO-TRPC1_EC_^−/−^ vs. DIO-TRPC1^fl/fl^ and DIO-TRPC1^fl/fl^ vs. CTL-TRPC1^fl/fl^ comparisons.

Building upon the dyslipidemia phenotype observed in endothelial TRPC1 deficiency, we conducted untargeted serum metabolomic profiling across four experimental groups: CTL-TRPC1_EC_^−/−^, CTL-TRPC1^fl/fl^, DIO-TRPC1_EC_^−/−^, and DIO-TRPC1^fl/fl^. Principal Component (PC) analysis revealed distinct metabolic clustering patterns among all groups (Figures 4A,E,I). Under basal conditions, a comparison between CTL-TRPC1_EC_^−/−^ and CTL-TRPC1^fl/fl^ indicated significant upregulation of (S)-homostachydrine and gibberellin A12 (GA12), while 1-methylhistidine and (1S, 2R)-1, 2-dihydrodibenzo [b, d]thiophene-1, 2-diol were markedly downregulated (Figures 4B,C). Pathway enrichment analysis suggested that perturbations in histidine metabolism represent a key metabolic signature (Figure 4D), indicating that TRPC1 deficiency may impair basal metabolic functions by disrupting amino acid homeostasis.

In the context of obesity, metabolomic analysis of DIO-TRPC1^fl/fl^ versus CTL-TRPC1^fl/fl^ mice revealed 294 significantly altered metabolites (Figure 4F; Supplementary Figure S4), implicating dysregulation across 10 metabolic pathways, including amino acid biosynthesis (Figure 4G). Venn diagram intersection analysis identified a persistent elevation of GA12 in both CTL-TRPC1_EC_^−/−^ and DIO-TRPC1^fl/fl^ groups, coupled with a consistent depletion of 1-methylhistidine (Figure 4H). Notably, while the role of GA12 as a phytohormone in mammalian systems remains poorly understood, our study demonstrates its pathological accumulation in TRPC1-deficient mice. This abnormal elevation may drive systemic inflammation (MacMillan et al., 1997; Biagioni et al., 2021). Conversely, 1-methylhistidine—a critical biomarker of muscle protein turnover—showed marked serum reductions in TRPC1-deficient mice (Kochlik et al., 2018). This depletion, which parallels observations in the visceral adipose tissue of metabolic syndrome patients, suggests impaired amino acid sensing and disrupted muscle protein synthesis (Piro et al., 2020). These findings strongly support the potential of 1-methylhistidine as a novel prognostic biomarker for obesity-associated metabolic complications.

Further analysis of endothelial TRPC1-specific deficiency under obese conditions (DIO-TRPC1_EC_^−/−^ vs. DIO-TRPC1^fl/fl^) identified five key downregulated metabolites, including N-acetylvaline, N-methyl-L-proline, indole-3-propionic acid, indole-3-acrylic acid, and beta-D-ethyl glucuronide (Figures 4J,K). Notably, N-acetylvaline exhibited a dual-phase downregulation (Figure 4L), suggesting that TRPC1 deficiency may further compromise metabolic performance through perturbations in amino acid biosynthesis (Holeček, 2020). Metabolic pathway network analysis (Figures 4D,G) revealed that the loss of TRPC1 synergistically suppresses amino acid biosynthesis and histidine metabolism, leading to the depletion of critical metabolites such as 1-methylhistidine and N-acetylvaline. This metabolic dysregulation exacerbates obesity-induced systemic metabolic dysfunction.

### 3.5 TRPC1 overexpression ameliorates obesity-induced metabolic dysfunction

**FIGURE 5:**
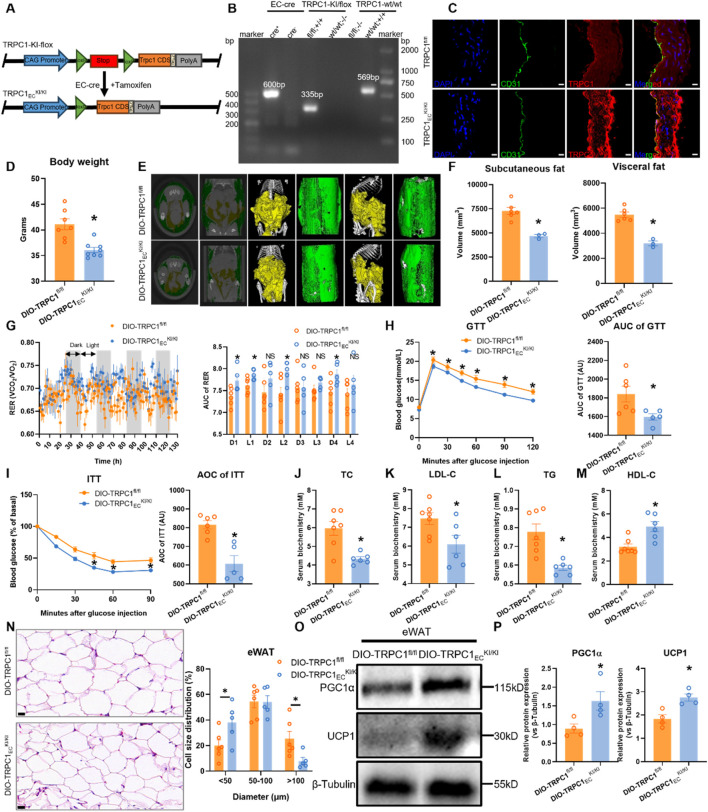
TRPC1 overexpression ameliorates obesity-induced metabolic dysfunction. **(A)** Schematic diagram illustrating the generation of TRPC1_EC_^KI/KI^ mice via EC-specific Cre-LoxP recombination. **(B)** Genotyping PCR validation of TRPC1_EC_^KI/KI^ and wild-type mice. **(C)** Immunofluorescence staining of thoracic aorta in TRPC1_EC_^KI/KI^ and TRPC1^fl/fl^ mice: nuclei (DAPI, blue), endothelial marker (CD31, green), and TRPC1 protein (red). Scale bar: 10 μm. **(D)** Body weight analysis of 20-week HFD-fed mice: DIO-TRPC1_EC_^KI/KI^ vs. DIO-TRPC1^fl/fl^ groups (n = 7–8/group). **(E)** Representative micro-CT images of adipose tissue distribution in DIO-TRPC1_EC_^KI/KI^ and DIO-TRPC1^fl/fl^ mice (subcutaneous fat: green; visceral fat: yellow). **(F)** Quantitative micro-CT analysis of subcutaneous and visceral fat volumes in both groups (n = 3–6/group). **(G)** RER dynamics in DIO-TRPC1_EC_^KI/KI^ and DIO-TRPC1^fl/fl^ mice, with AUC quantification (n = 5–6/group). **(H)** GTT (1.5 g/kg) results and AUC values for DIO-TRPC1^fl/fl^ and DIO-TRPC1_EC_^KI/KI^ mice (n = 5–6/group). **(I)** ITT (0.75 U/kg) results and AUC calculations for the same groups (n = 5–6/group). **(J–M)** Serum levels of TC, LDL-C, TG, and HDL-C in DIO-TRPC1^fl/fl^ and DIO-TRPC1_EC_^KI/KI^ mice (n = 6–7/group). **(N)** H&E staining of eWAT in both groups (scale bar = 20 μm) and size distribution pattern of adipocytes (n = 5–6/group). **(O,P)** Representative western blots and quantitative analysis of PGC1α and UCP1 protein levels in eWAT lysates from DIO-TRPC1^fl/fl^ and DIO-TRPC1_EC_^KI/KI^ mice (n = 4/group). *p < 0.05 vs. DIO-TRPC1^fl/fl^ group; NS: Not significant (*p* > 0.05).

The aforementioned findings demonstrate that endothelial TRPC1 deficiency exacerbates obesity-induced metabolic dysfunction. However, it remains unclear whether endothelial TRPC1 overexpression can mitigate this progression. To address this question, we generated a tamoxifen-inducible, endothelial-specific TRPC1-overexpressing mouse model (TRPC1_EC_^KI/KI^, Figure 5A), with successful EC-Cre recombination confirmed by genomic PCR analysis (Figure 5B). Immunostaining of thoracic aortas further validated TRPC1 protein overexpression in the endothelial cells of this model (Figure 5C). Evaluation of the metabolic protective effects of TRPC1 overexpression in HFD-induced obesity models revealed that TRPC1_EC_^KI/KI^ mice exhibited significant attenuation of weight gain following 12 weeks of HFD intervention (Figure 5D). Micro-CT reconstruction demonstrated marked reductions in both visceral and subcutaneous fat deposition compared to control groups (Figures 5E,F). Metabolic chamber monitoring data revealed elevated respiratory exchange ratios in obese TRPC1-overexpressing mice, suggesting improved efficiency in energy substrate utilization (Figure 5G), while basal metabolic parameters, including food intake, activity levels, and energy expenditure, remained unchanged (Supplementary Figure S5A–D). The TRPC1-overexpressing cohort demonstrated enhanced regulation of glucose homeostasis and insulin sensitivity (Figures 5H,I). Serum biochemical analysis revealed a significant improvement in lipid profiles, characterized by reduced pro-atherogenic lipid components and elevated protective lipoprotein levels (Figures 5J–M). Endothelial TRPC1 overexpression attenuates high-fat diet-induced epididymal white adipocyte hypertrophy, thereby decreasing the proportion of large adipocytes and increasing the proportion of small adipocytes within adipose tissue (Figure 5N), in parallel with upregulated expression of thermogenic proteins PGC1α and UCP1 (Figures 5O,P). Collectively, these findings indicate that endothelial TRPC1 overexpression ameliorates obesity-induced metabolic dysfunction.

## 4 Discussion

Dyslipidemia and glucose metabolism disorders induced by obesity represent significant public health challenges, with vascular endothelial cells acting as critical regulators of tissue microenvironmental homeostasis. The dysfunction of these cells is closely linked to the pathogenesis of metabolic diseases. Although the role of TRPC1—a key mediator of endothelial calcium signaling—in maintaining cardiovascular homeostasis is well-established, its functional mechanisms in metabolic regulation warrant further investigation. Utilizing an endothelial-specific TRPC1 knockout mouse model, this study reveals that TRPC1 deficiency, while not influencing the onset of obesity, exacerbates obesity-associated metabolic complications through the remodeling of the adipose tissue inflammatory microenvironment, impairment of thermogenic function, and disruption of serum metabolomic profiles. Conversely, TRPC1 overexpression exhibits significant metabolic protective effects by enhancing adipose tissue function and energy metabolism.

This study utilized male C57BL/6J mice for two primary reasons. First, female mammals demonstrate significant resistance to HFD-induced metabolic complications such as insulin resistance and nonalcoholic fatty liver disease, attributed to estrogen-mediated protective mechanisms (Acharya et al., 2021; Araujo et al., 2023). By focusing on males, we aimed to reduce confounding from inherent sexual dimorphism in metabolic susceptibility, aligning with conventional experimental designs in metabolic research (Krüger et al., 2020). Second, female rodent metabolic profiles are influenced by hormonal fluctuations during the estrous cycle, requiring increased sample sizes or prolonged study durations to control variance (Chari et al., 2020). The male model thus facilitated more efficient characterization of HFD-specific effects within our experimental constraints.

Despite comparable body weight gain and adipose tissue distribution in endothelial TRPC1-deficient mice subjected to high-fat diet intervention, these animals exhibit significant dysregulation of glucose and lipid metabolism. This phenotype suggests that TRPC1 deficiency may operate through a mechanism of ‘energy storage-utilization decoupling,’ wherein overall energy intake and fat deposition remain unaffected, but precise tissue-level energy utilization and partitioning are disrupted (Dahdah et al., 2021). As a calcium channel protein, TRPC1-mediated endothelial calcium signaling may regulate adipose tissue microvascular hemodynamics and vascular permeability, thereby influencing the efficiency of adipocyte lipid uptake and storage (Rode et al., 2019; Schaar et al., 2019). Although respiratory metabolic monitoring reveals only a marginal decline in thermogenesis, these findings imply that TRPC1 may subtly modulate mitochondrial function or thermogenic pathways, thereby influencing energy metabolism (Benzi et al., 2023). Such subthreshold effects could progressively accumulate under chronic metabolic stress, ultimately precipitating tissue dysfunction.

This study observed that endothelial TRPC1 deficiency specifically induces hypertrophy of eWAT without significantly affecting inguinal white adipose tissue or brown adipose tissue. This depot-specific effect may arise from the molecular and functional heterogeneity present in vascular endothelial cells across different adipose depots (Wei et al., 2024). As a metabolically active visceral fat, eWAT hypertrophy can compromise lipid droplet stability, increase free fatty acid efflux, and promote ectopic lipid deposition in hepatocytes and skeletal muscle, thereby disrupting insulin signaling (Morais et al., 2019).

Concurrently, the hypoxic microenvironment associated with adipocyte hypertrophy may activate endothelial pro-inflammatory responses, enhancing the secretion of chemokines such as CCL3 and CXCL5. This process facilitates the recruitment of immune cells, including macrophages, thereby establishing a chronic inflammatory microenvironment (Tourniaire et al., 2013; van Poppel et al., 2018). Such localized inflammation impacts systemic metabolism through paracrine mechanisms; for instance, the pro-inflammatory cytokine IL-1β suppresses the expression of the thermogenic gene UCP1 in adipocytes, which reduces energy expenditure and exacerbates insulin resistance. Notably, the downregulation of PGC1α and UCP1 in eWAT not only reflects impaired thermogenic function but may also indicate the inhibition of white adipose tissue browning. Endothelial TRPC1 likely participates in this process by modulating vascular endothelial growth factor (VEGF) and other mediators, thereby influencing the microvascular density and oxygen supply of adipose tissue (Lee, 2018).

Endothelial TRPC1 deficiency induces depot-specific inflammatory responses. This regional heterogeneity likely reflects differences in vascular endothelial cell function and immune cell composition across adipose depots (Vijay et al., 2020). In visceral fat depots with dense microvascular networks, TRPC1 deficiency may enhance chemokine expression through NF-κB pathways, promoting recruitment of pro-inflammatory M1 macrophages and establishing a pro-inflammatory microenvironment (Lin et al., 2024). Conversely, compensatory elevation of anti-inflammatory factors in brown fat and perivascular adipose tissue may represent systemic negative feedback regulation, though this response fails to effectively counteract concurrent pro-inflammatory cytokine upregulation (e.g., IL-1β), indicating a dysregulated inflammatory network (Ding et al., 2016). This immunometabolic disruption not only directly impairs adipocyte function but also compromises vascular endothelial-dependent relaxation, creating a vicious cycle of “adipose-vascular” cross-talk dysfunction that exacerbates systemic metabolic dysregulation (Pelham et al., 2016).

Serum metabolomic analysis revealed that endothelial TRPC1 deficiency causes significant depletion of 1-methylhistidine, a key metabolite in the histidine metabolism pathway. As a biomarker of muscle protein turnover, reduced 1-methylhistidine levels may reflect attenuated skeletal muscle catabolism, contributing to obesity-related muscle dysfunction and amino acid sensing abnormalities (Davydova et al., 2021). Notably, the plant-derived hormone GA12 shows pathological accumulation in TRPC1-deficient mice. While its physiological roles in animals remain undefined, GA12 may mediate systemic inflammation via NF-κB pathway activation, potentially serving as a mechanistic link between endothelial dysfunction and metabolic dysregulation (Reihill et al., 2016). Additionally, marked downregulation of N-acetylvaline under obese conditions suggests TRPC1 may modulate mitochondrial energy metabolism through amino acid biosynthesis regulation (Sharma et al., 2024). This aligns with observed reductions in thermogenic gene expression and respiratory exchange ratios, collectively indicating TRPC1’s profound regulatory role in energy substrate utilization.

Gain-of-function experiments confirm that the overexpression of endothelial TRPC1 significantly ameliorates obesity-associated phenotypes in mice. These findings not only validate the phenotypic specificity of TRPC1 deficiency but also suggest that TRPC1 may enhance vascular endothelial function to improve the microvascular architecture and blood perfusion of adipose tissue, thereby establishing a metabolically favorable microenvironment for adipocytes (Lear et al., 2015). Notably, elevated respiratory exchange ratios in TRPC1-overexpressing mice indicate a beneficial shift in energy substrate utilization from lipids to carbohydrates, potentially linked to TRPC1-mediated enhancement of mitochondrial biogenesis and oxidative metabolism (Ding et al., 2023). Collectively, these results provide a robust experimental rationale for targeting TRPC1 in the therapeutic development of metabolic disorders.

TRPC1 deficiency directly reduces endothelial Ca^2+^ influx, leading to impaired endothelial nitric oxide synthase (eNOS) phosphorylation and diminished nitric oxide (NO) production (Zhu et al., 2025). This NO deficit disrupts the soluble guanylate cyclase (sGC)/Protein Kinase G Iα (PKGIα)/Glycogen Synthase Kinase 3β (GSK3β) axis critical for perivascular progenitor cell (PPC)-to-BAT differentiation (Park et al., 2022), resulting in reduced BAT mass, impaired thermogenesis, and systemic energy imbalance, while promoting ectopic lipid storage. Concurrently, endothelial dysfunction impairs insulin-stimulated glucose disposal, contributing to insulin resistance (Geng et al., 2021). Additionally, TRPC1 ablation induces mitochondrial Ca^2+^ overload and dynamics dysregulation, compromising ATP synthesis (Wang et al., 2018) and exacerbating ROS-driven oxidative stress (He et al., 2016). These metabolic perturbations foster a proinflammatory milieu. Furthermore, TRPC1 deficiency upregulates c-Fos (Zhu et al., 2025), which drives inflammatory gene transcription via AP-1 and MAPK pathway activation (Yang et al., 2025), while interacting with NF-κB to amplify inflammatory cascades (Kim et al., 2024). Collectively, these multilayered mechanisms synergize to exacerbate obesity and adipose tissue inflammation.

Notably, this study did not address whether TRPC1 function or localization depends on lipid raft integrity—a critical gap given these domains’ roles as signaling platforms for endothelial proteins. Lipid rafts, enriched in cholesterol and sphingolipids, are essential for organizing membrane receptors and modulating pathways like Wnt/β-catenin signaling and inflammation (Riitano et al., 2020). For example, anti-β2-GPI antibodies recruit LRP6/PAR-2 complexes into rafts to induce prothrombotic responses (Riitano et al., 2022), while raft dynamics regulate vascular function in metabolic disease through protein compartmentalization (Riitano et al., 2023). Future studies using lipid raft disruptors (e.g., MβCD) could clarify whether TRPC1 resides in or requires these microdomains for its metabolic actions, with implications for therapeutic targeting.

This study unveils endothelial TRPC1 as a critical regulator of metabolic homeostasis, with its dysfunction driving obesity-related metabolic complications through a cascade of “adipose tissue inflammation - thermogenic failure - metabolomic disruption.” However, the precise molecular pathways through which TRPC1 modulates vascular-immune interactions in adipose tissue, as well as the metabolic fate of GA12 in animals, warrant further investigation. Future studies could employ single-cell sequencing to dissect endothelial cell heterogeneity across adipose depots, thereby clarifying TRPC1’s cell-specific functions. Concurrently, clinical validation of metabolites such as 1-methylhistidine as biomarkers for obesity-associated metabolic dysfunction holds significant promise. In summary, this work provides novel insights into the role of vascular endothelial cells in metabolic diseases and identifies TRPC1 as a highly valuable therapeutic target for intervention.

## Data Availability

The original contributions presented in the study are included in the article/Supplementary Material, further inquiries can be directed to the corresponding author.
