# Supplementary material for: Loss of endothelial TRPC1 aggravates metabolic dysfunction in obesity via disrupting adipose tissue homeostasis
Source: Front Mol Biosci. 2025 Jun 11;12:1619559. doi: 10.3389/fmolb.2025.1619559 (PMC12187605; doi:10.3389/fmolb.2025.1619559)
Supplement: Supplementary file 1 [file Supplementaryfile1.docx]

# **Loss of endothelial TRPC1 aggravates metabolic dysfunction in obesity via disrupting adipose tissue homeostasis**

Yihui Lan^1^, Shiquan Wang^1^, Yuan Chu^1^, Yizhi Zhang^1^, Yuan Liu^1^, Fan Yu^1^, Lei Feng^1^, Yifei Zhu^1*^

^1^ Wuxi School of Medicine, Jiangnan University, 1800 Lihu Road, Wuxi, China.

^*^ Corresponding author.

E-mail address: [yifeizhu@jiangnan.edu.cn](mailto:yifeizhu@jiangnan.edu.cn) (Y. Zhu).

# **SUPPLEMENTARY TABLES**

**SUPPLEMENTARY TABLE 1** Primer List for Mouse Genotyping.

| Gene name | Forward primer（5’-3’） | Reverse primer（5’-3’） |
| --- | --- | --- |
| EC-cre | CGCATAACCAGTGAAACAGCATTGC | CCCTGTGCTCAGACAGAAATGAGA |
| TRPC1-flox | CTTCTCCTTCCAGTTTCCACTCTG | CTACTTGATCTACCAGGAGCTGGC |
| TRPC1-KI-flox-Targeted | GGGCAGTCTGGTACTTCCAAGCT | TGGCGTTACTATGGGAACATACGTC |
| TRPC1-KI-flox-WT | CAGCAAAACCTGGCTGTGGATC | ATGAGCCACCATGTGGGTGTC |

**SUPPLEMENTARY TABLE 2** PCR amplification protocol for TRPC1-flox

| Cyclic steps | Temperature | Time | Cyclic number |
| --- | --- | --- | --- |
| 1 | 95℃ | 5 min |  |
| 2 | 98℃ | 30 sec | 20 |
| 3 | 65℃(-0.5℃/ cycle) | 30 sec |  |
| 4 | 72℃ | 45 sec |  |
| 5 | 98℃ | 30 sec |  |
| 6 | 55℃ | 30 sec | 20 |
| 7 | 72℃ | 45 sec |  |
| 8 | 72℃ | 5 min |  |
| 9 | 10℃ | hold |  |

**SUPPLEMENTARY TABLE 3** PCR amplification protocol for EC-cre

| Cyclic steps | Temperature | Time | Cyclic number |
| --- | --- | --- | --- |
| 1 | 94℃ | 3 min |  |
| 2 | 94℃ | 30 sec | 35 |
| 3 | 60℃ | 30 sec |  |
| 4 | 72℃ | 30 sec |  |
| 5 | 72℃ | 30 sec |  |
| 6 | 10℃ | hold |  |

**SUPPLEMENTARY TABLE 4** PCR amplification protocol for TRPC1-KI-flox

| Cyclic steps | Temperature | Time | Cyclic number |
| --- | --- | --- | --- |
| 1 | 95℃ | 5 min |  |
| 2 | 98℃ | 30 sec | 20 |
| 3 | 68℃(-0.5℃/ cycle) | 30 sec |  |
| 4 | 72℃ | 45 sec |  |
| 5 | 98℃ | 30 sec | 12 |
| 6 | 58℃ | 30 sec |  |
| 7 | 72℃ | 45 sec |  |
| 8 | 72℃ | 5 min |  |
| 9 | 10℃ | hold |  |

**SUPPLEMENTARY TABLE 5** List of primers used for q-PCR.

| Gene name | Forward primer（5’-3’） | Reverse primer（5’-3’） |
| --- | --- | --- |
| Adiponectin | GCACTGGCAAGTTCTACTGCAA | GTAGGTGAAGAGAACGGCCTTGT |
| ADRβ3 | GGCCCTCTCTAGTTCCCAG | TAGCCATCAAACCTGTTGAGC |
| CIDEA | TGCTCTTCTGTATCGCCCAGT | GCCGTGTTAAGGAATCTGCTG |
| COX4I1 | ACCAAGCGAATGCTGGACAT | GGCGGAGAAGCCCTGAA |
| COX8b | GAACCATGAAGCCAACGACT | GCGAAGTTCACAGTGGTTCC |
| Leptin | GAGACCCCTGTGTCGGTTC | CTGCGTGTGTGAAATGTCATTG |
| PGC1α | CCCTGCCATTGTTAAGACC | TGCTGCTGTTCCTGTTTTC |
| PGC1β | GGCAGGTTCAACCCCGA | CTTGCTAACATCACAGAGGATATCTTG |
| PPARγ | GTGCCAGTTTCGATCCGTAGA | GGCCAGCATCGTGTAGATGA |
| UCP1 | ACTGCCACACCTCCAGTCATT | CTTTGCCTCACTCAGGATTGG |
| IL-4RA | TGGATCTGGGAGCATCAAGGT | TGGAAGTGCGGATGTAGTCAG |
| SOCS3 | ATGGTCACCCACAGCAAGTTT | TCCAGTAGAATCCGCTCTCCT |
| SOCS5 | GAGGGAGGAAGCCGTAATGAG | CGGCACAGTTTTGGTTCCG |
| TIMP1 | GCAACTCGGACCTGGTCATAA | CGGCCCGTGATGAGAAACT |
| TNF-α | TCGAGTGACAAGCCTGTAGCC | TTGAGATCCATGCCGTTGG |
| IL-10 | CTTACTGACTGGCATGAGGATCA | GCAGCTCTAGGAGCATGTGG |
| CD68 | TTCTCCAGCTGTTCACCTTGACCT | GTTGCAAGAGAAACATGGCCCGAA |
| CCL3 | TTCTCTGTACCATGACACTCTGC | CGTGGAATCTTCCGGCTGTAG |
| CCL7 | GCTGCTTTCAGCATCCAAGTG | CCAGGGACACCGACTACTG |
| CCL8 | TCTACGCAGTGCTTCTTTGCC | AAGGGGGATCTTCAGCTTTAGTA |
| CXCL1 | CTGGGATTCACCTCAAGAACATC | CAGGGTCAAGGCAAGCCTC |
| CXCL5 | TGCGTTGTGTTTGCTTAACCG | AGCTATGACTTCCACCGTAGG |
| SAA3 | TGCCATCATTCTTTGCATCTTGA | CCGTGAACTTCTGAACAGCCT |
| IL-1β | TCGCTCAGGGTCACAAGAAA | CCTCTCGATGACGAAGC |
| TBP | GAAGCTGCGGTACAATTCCAG | CCCCTTGTACCCTTCACCAAT |

# **SUPPLEMENTARY FIGURES**


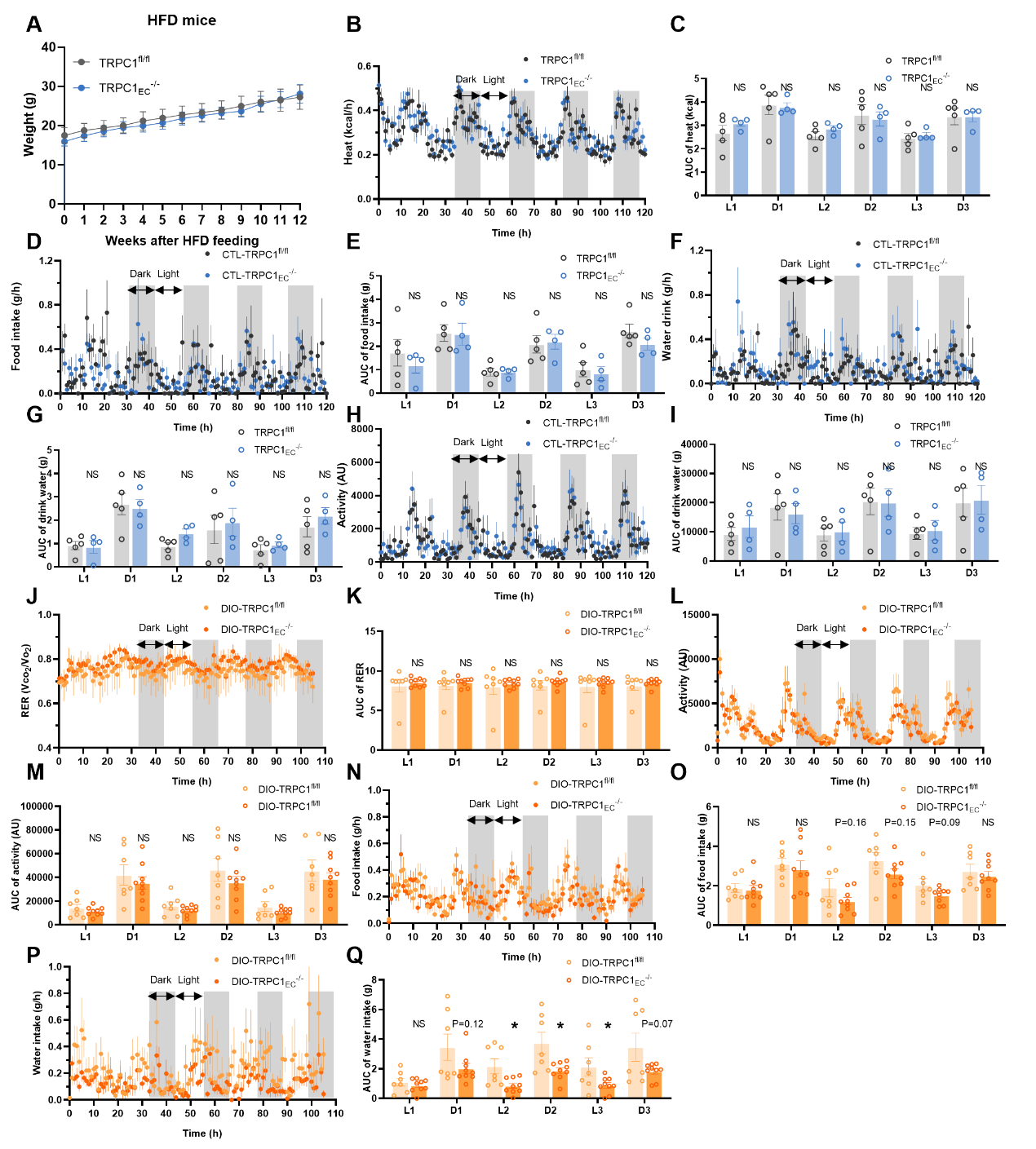


**SUPPLEMENTARY FIGURE 1**

Endothelial cell-specific TRPC1 deficiency did not significantly affect respiratory exchange ratio (RER) or physical activity levels. **(A)** Longitudinal monitoring of body weight changes in high-fat diet (HFD)-fed mice from 8 to 20 weeks of age. **(B–I)** Measurements of heat consumption, food intake, water intake, and activity levels in CTL-TRPC1^fl/fl^ and CTL-TRPC1_EC_^−/−^ mice, with data calculated and presented as area under the curve (AUC) values for each experimental phase. **(J–Q)** Monitoring of RER, activity, food and water intake in DIO-TRPC1^fl/fl^ and DIO-TRPC1_EC_^−/−^ mice, along with AUC quantification for each parameter. NS: No statistical significance (*p* > 0.05).


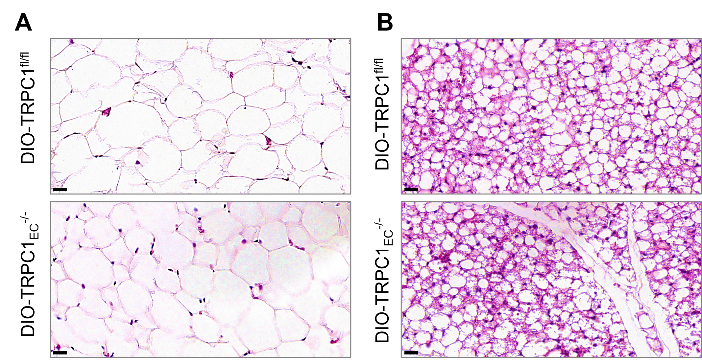


**SUPPLEMENTARY FIGURE 2**

Endothelial cell-specific TRPC1 deficiency in obese mice did not significantly alter adipocyte lipid droplet size in inguinal white adipose tissue (iWAT) and brown adipose tissue (BAT). **(A–B)** Representative hematoxylin and eosin (H&E) staining of iWAT and BAT from DIO-TRPC1^fl/fl^ and DIO-TRPC1_EC_^−/−^ mice. Scale bar: 20 μm.


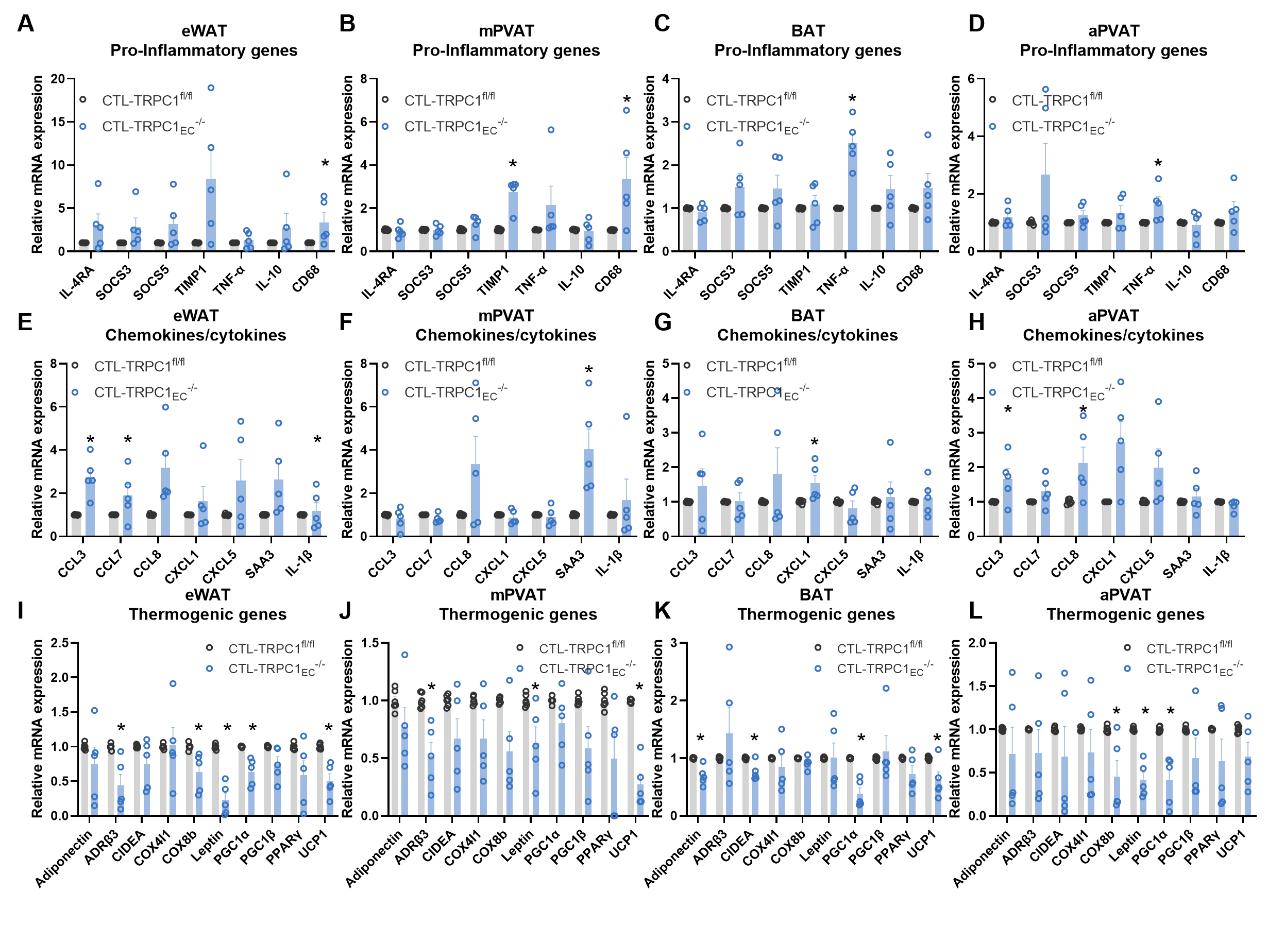


**SUPPLEMENTARY FIGURE 3**

Endothelial TRPC1 deficiency exacerbates adipose tissue inflammation and impairs thermogenic gene expression. **(A–D)** Relative mRNA expression of pro-inflammatory genes in eWAT, mesenteric perivascular adipose tissue (mPVAT), brown adipose tissue (BAT), and aortic perivascular adipose tissue (aPVAT) from CTL-TRPC1^fl/fl^ and CTL-TRPC1_EC_^−/−^ mice. **(E–H)** Relative mRNA levels of chemokines/cytokines in the same tissues and mouse groups. **(I–L)** Relative mRNA expression of thermogenic genes in indicated tissues.


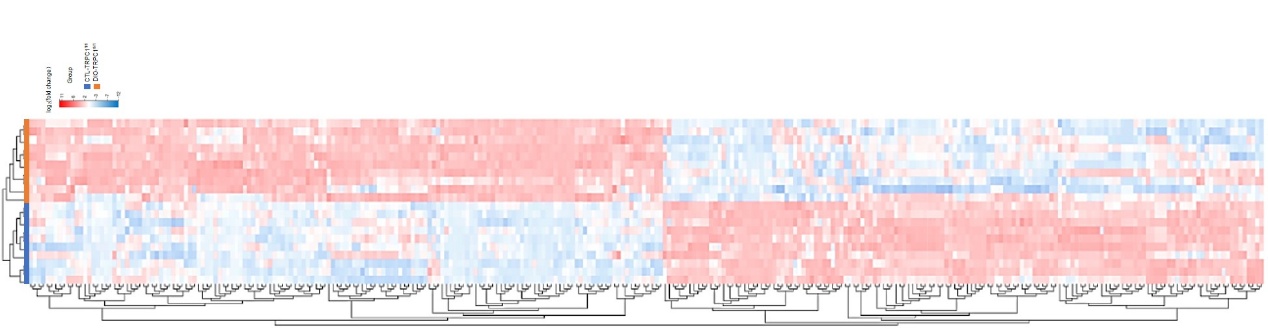


**SUPPLEMENTARY FIGURE 4**

**(A)** Correlation heatmap comparing serum metabolite profiles between CTL-TRPC1^fl/fl^ and DIO-TRPC1^fl/fl^ mice.


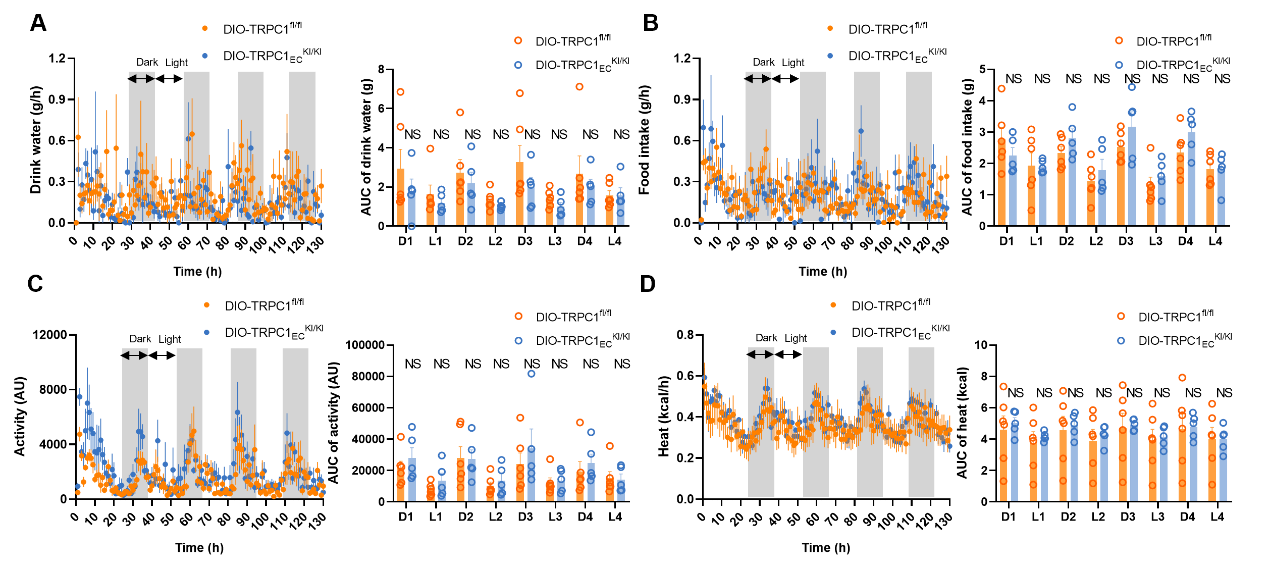


**SUPPLEMENTARY FIGURE 5**

Endothelial cell-specific TRPC1 overexpression did not significantly alter water intake, food intake, activity, or heat. **(A–D)** Measurements of water intake, food intake, activity, and heat in DIO-TRPC1^fl/fl^ and DIO-TRPC1_EC_^KI/KI^ mice, with AUC values calculated for each parameter. NS: No statistical significance (*p* > 0.05).
